# The patient’s view on rare disease trial design – a qualitative study

**DOI:** 10.1186/s13023-019-1002-z

**Published:** 2019-02-07

**Authors:** C. M. W. Gaasterland, M. C. Jansen – van der Weide, M. J. du Prie – Olthof, M. Donk, M. M. Kaatee, R. Kaczmarek, C. Lavery, K. Leeson-Beevers, N. O’Neill, O. Timmis, V. van Nederveen, E. Vroom, J. H. van der Lee

**Affiliations:** 1 Pediatric clinical Research Office, Academic Medical Center, University of Amsterdam, Meibergdreef 9, 1105 AZ Amsterdam, The Netherlands; 2 Department of Cognitive Psychology, Vrije Universiteit Amsterdam, Amsterdam, the Netherlands; 3 PSC Patients Europe, Bennebroek, the Netherlands; 4 Laboratory of Glycoconjugate Immunochemistry, Hirszfeld Institute of Immunology and Experimental Therapy, Polish Academy of Sciences, Wroclaw, Poland; 5 Polish Hemophilia Society, Warsaw, Poland; 6 MPS Society, Amersham, UK; 7 Alström Syndrome UK, Torquay, Devon UK; 8 EUPATI Fellow, Bennebroek, the Netherlands; 9 AKU Society, Cambridge, UK; 10 Stichting Patiëntenstem.nu, Den Haag, The Netherlands; 11 Duchenne Parent Project, Veenendaal, The Netherlands

**Keywords:** Rare diseases, Patient involvement, Qualitative study

## Abstract

**Background:**

Clinical trials in rare diseases are more challenging than trials in frequent diseases. Small numbers of eligible trial participants, often complicated by heterogeneity among rare disease patients, hamper the design and conduct of a ‘classical’ Randomized Controlled Trial. Therefore, novel designs are developed by statisticians. However, it is important to be aware of possible design aspects that may jeopardize the feasibility of trial conduct. If the burden of participation is considered out of proportion by patients or parents, recruitment may fail or participants may drop out before trial completion. In order to maximize the chance of success of trials in small populations, it is important to know which aspects of trial design are considered important by patients.

**Results:**

We have interviewed all ten members of the Patient Think Tank (PTT) of the ASTERIX project, a European research consortium on methodology for clinical trials in small populations. The PTT members are rare disease patient representatives who have completed extensive training in clinical trial methodology. We have analyzed the interviews qualitatively according to Grounded Theory using a thematic analysis, and we structured the topics in four chronologically ordered themes: 1. Involvement in trial design; 2. Opinions on trial design; 3. Trial participation; 4. Phase after the trial. Our main findings are that the PTT-members recommend that patients are involved in trial design from an early stage on, and have influence on the outcomes and measurement instruments that are chosen in the trial, the length of the study, the choice of participants, and the information that is sent to potential participants. Also, according to the PTT-members, patient groups should consider setting up disease registries, placebo groups should be minimized, and more education on clinical trials is advised.

**Conclusions:**

Rare disease patient representatives who have been educated about clinical trial methodology think it is important to involve patient representatives in research at an early stage. They can be of advice in trial design in such a way that the ratio of potential benefit and burden of trial participation as well as the chosen outcome measures and in- and exclusion criteria are optimized.

**Electronic supplementary material:**

The online version of this article (10.1186/s13023-019-1002-z) contains supplementary material, which is available to authorized users.

## Introduction

A disease is defined as rare when the prevalence is lower than one in 2000 individuals in the European Union [1]. However, the total number of people suffering from a rare disease is large. It is estimated that between 5000 and 7000 rare diseases exist. Thus, the proportion of individuals with a rare disease is estimated at 6 to 8% of the population in the EU, according to the European Organization for Rare Diseases [2]. Most rare diseases are associated with unmet needs due to a lack of available treatments and the relative lack of research to discover and develop such treatments, and consequently a high socio-economic burden [3].

Because of this high burden, health authorities decided upon regulatory incentives for Orphan Medical Products, which led to an increase in the number of applications for market authorization of orphan drugs. However, trials in rare diseases are more challenging than clinical trials in more frequent diseases. The classical randomized controlled trial (RCT) cannot always be conducted, because of small numbers of eligible patients and the heterogeneity of the patient groups. Patients may be in various stages of a disease and disease courses may be far from uniform. When an RCT is conducted, it may be subject to imperfections in practical conduct, inclusion criteria, and trial design [4]. In recent years, new clinical trial methods have been developed that promise to be more efficient than classical RCTs [5–8].

When it comes to maximizing the chance of a successful trial, the patients or parents of a child with a rare or ultra-rare disease can play a pivotal role when they are involved in an early stage. Although there is no structural evidence that patient involvement improves the efficacy of trials [9], there are some examples where patient involvement has made a difference [10, 11]. If the burden of participation is considered to be out of proportion, recruitment may fail or participants may drop out before trial completion. Early involvement of patient representatives can help identify design aspects that may render a trial acceptable for potential participants. By removing or adapting undesired design aspects the chance of success of a trial may be enhanced. Patients can also assist with the choice of informative and relevant outcome measures [12]. The role of the patient organizations has consequently become more important in terms of funding research and setting the research agenda [13–15].

One example where the activity of the patient community has had considerable influence on the research field is Hemophilia. This collaboration resulted in the implementation of quality initiatives that directly improved hemophilia patient care [16]. Patients have changed the field and have become equal partners in the discussion on treatment policy. They are active in setting the research agenda, and in the debate on which outcome measures should be included in studies [17].

In order to investigate the patients’ views on clinical trial design, we conducted a qualitative study in a selected group of 10 educated rare disease patient representatives. Our main question was: Which aspects of trial design are relevant to you, and what is your opinion on these topics?

## Methods

For this research, we have followed the Standards for Reporting Qualitative Research (SRQR) and the Consolidated criteria for reporting qualitative research (COREQ) guidelines [18, 19] For the analysis a thematic analysis was used [20].

### Participants and recruitment process

Ten patient representatives were interviewed, who were all members of the Patient Think Tank (PTT) of a European research consortium for methodology of rare disease trials called ASTERIX [21]. The PTT-members have been chosen to represent a wide range of rare disease categories, varying from acute to chronic diseases. They had all been trained extensively in clinical trial design and regulatory issues, either by the EURORDIS summer school, EUPATI courses or through at least ten years of experience. Some of the PTT-members have a rare disease themselves, whereas others are parent of a child with a rare disease. One of the PTT-members is a professional representative of patients with a particular rare disease. Three of the ten PTT-members are male. The PTT-members live in various countries in the EU: the Netherlands, Poland, Germany, the UK and Ireland. The selection of participants was a convenience sample: it was an opportunity to be able to speak to this variety of persons involved in rare diseases as they were all part of the ASTERIX project. In this capacity, the participants, being PTT-members, knew the reason and objectives of the study.

### Data collection

The PTT-members were approached by the patient contact person of ASTERIX and were asked if they would be interested in participating in an interview about their experience with rare disease research, and what aspects of trial design they would perceive as important. All ten PTT-members agreed to participate and gave their oral consent. The interviews were held between May 18th and July 17th 2015, by videoconference. Each participant was interviewed once by two (out of three) interviewers (CG, MJW, and MdP). All interviewers followed a two day course on qualitative interviewing prior to the research, and had some previous experience in conducting qualitative interviews. Of the ten interviews, six were conducted in English, and four in Dutch. For the interviews the program GoToMeeting was used, which provides the possibility for video-conference. All interviews were recorded, for which the participants also gave their oral consent. Each interview was conducted according to a pre-set Interview Guide (see Additional file 1). The median interview duration was 53 min, with a range of 41 to 69 min.

### Data analysis

The verbatim transcript of every interview was sent to the respective participant to give the participants the opportunity to remove quotes that they did not want to appear in the final manuscript. For the analysis, the program MaxQda was used. A code tree was developed, based on the interview questions and the main topics that emerged, to structure the interview coding process. As a means to achieve reliable coding, transcripts were coded by the first author (CG) and checked by the second author (MJW). Several times, the researchers met to discuss the coding. Any discrepancies were resolved by discussing the codes, until consensus was reached. The codes were then grouped into conceptual categories, and a summary of all quotes within each conceptual category was made. The interrelationships between the categories were discussed between the coders, and a clear structure for the manuscript was made. The resulting manuscript was sent to all participants to check whether the topics that were most relevant to the participants were presented appropriately.

## Results

We present the themes that arose from the interviews according to the chronology of the design development and conduct of a clinical trial; the first theme is Involvement in trial design, the second theme is Opinions on trial design, the third theme is Trial participation, and the last theme is Phase after the trial.

### Theme 1: Involvement in trial design

#### Heterogeneity of patient groups

For many rare diseases, a patient organization or patient group exists. When patients are organized, they have a better chance at collaborating with researchers and being able to influence which direction research is taking. In research, there are many different stakeholders: patients, patient representatives, researchers, pharmaceutical industry, regulators. The members of the PTT mentioned that often, the initiator of a drug trial is a pharmaceutical company, but there are exceptions. For example, some of the PTT-members mentioned that with national or European funding, the patient group initiated a trial with help of a company, instead of the other way around.

There are many differences within patient organizations; it is important not to generalize. For example, one of the PTT-members mentioned that within patient organizations age is an important factor in how patients feel about research and which direction the research should take. Parents of young children with a particular disease often have higher expectations of the chance to find a cure than parents of older patients, who have tempered their expectations, and may be looking for small improvements in symptoms. One member of the PTT mentioned that there may also be cultural differences between countries that affect the willingness to participate in a pharmaceutical trial.

***Quote P4:*** *‘..because if I asked a group of 100 [disease] patients: If you had the choice, would you choose The Cure or quality of life?’, I am not sure what they would choose. I should ask that question sometime. I think it would be 50/50. I also think that age is a relevant factor. There are also children with [disease]. Not as many, fortunately, but they do exist. And yes, I think they, and their parents, would choose The Cure because they still have a whole life ahead of them. While you - I don’t know how old you are - when you’re a bit older, usually it is diagnosed around the age of 40, may consider it differently.’*

***Quote P1:*** *‘in France for instance, it’s really different, they’re much more open to trials, clinical trials, there is a lot of reservedness, is that the right word? I don’t know.. people here are very reserved when it comes to pharmaceutical companies.. as I said, in other countries it’s much different.”*

#### Collaboration between patients and researchers

Some of the PTT-members mentioned that the patient organizations in which they are involved have initiated one or more trials, whereas other PTT-members felt that their patient organizations were not involved in the setup of a trial early enough. These patient organizations were only approached when the trial was already recruiting, as a source of participants. Most PTT-members preferred patient organizations to be involved in the trial at an early stage.

***Quote P7:*** *‘you know, we’re funded by [national funding agency], in this partnership with the center of excellence. So because we’ve already got that set up, we have got that relationship with the leads in our team from the children’s and the adult’s center, that they already liaised with us closely anyway. We are.. you know, on a contract with [..] children’s hospital for example, so I’m fully involved in their clinic. So I’m present at every children and adult clinic. So because I am involved to that extent it is much easier for me to be involved in any.. you know, any kind of trial. Because they.. you know, the doctors and myself liaise quite regularly anyway. For other patient organizations that aren’t set up in that way I can see where that would be much more difficult. And, you know, and we fought for a long time, as a patient organization, to be recognized as equal partners in the survey. So it’s not been an easy road. But I think it’s.. you know, we’ve done all the ground work to get to where we are now.’*

***Quote P9:*** *‘One company.. well several companies were coming to us at the pre-clinical stage to discuss their trial design because we have a lot of knowledge in our team. And we’ve also facilitated patient workshops where the patients or their carers have input into the design in looking at the quality of life measures and how appropriate they are.’*

***Quote P1:*** *‘Well.. Yes, in the trials design, we were not involved. We were.. well at least from [pharmaceutical company] side, we were involved at a relatively early stage, but I think the background really was to try to find enough patients to participate, because that is of course the problem in a rare disease, you just can’t find enough patients.. enough participants, in trials..’*

#### Collaboration between patients and industry

Many PTT-members mentioned the presence of collaboration between their patient organization and the pharmaceutical industry. These collaborations have led to very fruitful results, according to the PTT-members, but also posed some problems, such as difficulties in communication between patients and companies due to conflicting interests, i.e., health versus commercial interests. Some of the PTT-members mentioned this, and an example is given below:

***Quote P1:*** *‘But what we did was we gave the companies time, and time slots in our conferences, in order to inform about the status, which was again a problem, because they said well, there is nothing published, and we know more than we are allowed to say, there is of course also law problems with that, but.. sometimes I thought it’s also just because of.. there was a competition going on between at least these two companies, and they just didn’t want to tell more’*

### Theme 2: Opinions on trial design

#### Outcome measures

One of the concrete topics that the PTT-members feel they should be involved in, is the choice of clinically meaningful outcome measures. They argue that patients have the most hands-on knowledge of which outcomes are relevant form a patient perspective and should be added in the study.

***Quote P2:*** *‘Yeah, I suppose that’s the key thing really, is making sure that patients have the chance to give their views, and then that those views are listened to. And.. kind of more practical things. They.. the patients wanted to make sure that.. the kind of outcomes were sort of relevant in their life, so, you know, the idea of looking beyond just the clinical outcomes’*

#### Length of a study

Also, PTT-members mentioned that some of the trials that they were involved in were too short to show an effect of the intervention. It is possible that companies want shorter trials, where patients want a longer intervention period to make sure that a possible effect of the intervention is shown. They want to be involved in decisions about the duration of the study.

***Quote P1:*** *‘But.. the problem of course is still the.. Well, the whole, the duration of the trial, as I said, was not long enough, and it started too late. They knew this, right, they knew they.. should have applied this medication earlier, and.. but they could.. just couldn’t do it because of laws’*

#### Inclusion criteria for participation in a trial

One of the PTT-members mentioned the decision on inclusion criteria for the trial. The PTT-member (a parent of a patient) felt that the efficacy of a particular drug could only be demonstrated when it was applied in children. This was problematic, since most drugs have to be tested in adults before they can be tested in children. The PTT-member would have preferred to be involved in this part of the trial design.

***Quote P1:*** *‘the other thing that happened was that they had started with adults. The brain development, of course, is going on much earlier. And.. So you should have really started at a very young age. But then, you can’t do this, yeah? You can’t start with a newborn.. And try to influence the missing protein pathway.. because it’s just too dangerous. And to.. even if you can prove that it doesn’t harm you, in an adult, that doesn’t mean that it doesn’t harm harm a newborn. Whereas you really needed to apply this medication to a newborn in order to influence the development of the brain properly. So this is really a difficult situation, or was a difficult situation, and both trials ended unsuccessfully last year.’*

#### Use of placebo arm and randomization

In many trials, an experimental drug is compared with a placebo in addition to the standard of care. Many of the PTT-members would want to decrease the chance of being allocated to the placebo arm of a trial as much as possible. The new intervention that is tested is perceived as a new possible treatment, and patients are hopeful that they may benefit from it; it may be their only source of hope. However, it was also mentioned that not all participants in a trial prefer to be in the experimental arm:

***Quote P2:*** *‘the one [potential trial participant] particularly was unhappy because she wanted to have a baby, and obviously if you’re taking the treatment, then you try is not to get pregnant. And so she was hoping that she’d be in the no treatment arm and then she could have a baby during the trial. So, yeah, I mean, it kind of worked both ways I guess.’*

Many of the PTT-members mentioned that ‘fair use of placebo’ would be a good option. This means that every participant in a study gets an equal share of placebo and experimental drug, such as in a cross-over study. Also, options for making the placebo group as small as possible were mentioned, such as sharing placebo data over trials or using natural history data as comparator.

***Quote P8:*** *‘..what I find extraordinary is that the company for example, also because we requested them to, shared their placebo data with other companies. So you don’t.. because.. we of course think it’s a waste.. all those children who are in a placebo group. The smaller the placebo group, the better.’*

Several PTT-members notified us that trial participants sometimes do not completely grasp the consequences of randomization. They hope that they are allowed to influence to which trial arm they are allocated, or are disappointed when they are assigned to the arm that did not have their preference. According to the PTT-members, many patients have a clear preference when they enter a trial.

***Quote P2:*** *‘And that was a question that always came back, that people knew that it was a 50-50 chance, but they wanted to somehow push it into their favor, and we just had to say they couldn’t do that.’*

#### Registries

In our interviews, the topic of disease registries was something that was discussed regularly. A PTT-member gave an example of how setting up a strong patient registry would have been a great way of avoiding the use of a placebo in a trial, if complete natural history data is accepted as a comparison of experimental drug use.

***Quote P2:*** *‘Ideally, we would have had a strong patient registry. So if I could go back in time we would have set up a really strong patient registry. We would have followed up, you know, maybe a hundred patients, and followed them up for five years, properly recorded, every, you know, medical change that happened to them, done properly a sort of near observational study monitoring on those patients. And then, once we were ready to do a clinical trial, we would have then done this prospective study, so then it would have meant that all patients were taking the drug, and that we were comparing them to their past selves. That would have been absolutely fantastic. It would have solved a lot of issues that.. all patients would then be on the treatment, and then there would be no no-treatment arm. That would have been fantastic, but.. you know obviously, we haven’t done that. But that would have been a great way of doing it, I think.’*

#### Should patients be involved in all topics?

Not all topics of trial design are considered relevant for patients by the members of the PTT. One of the members stated that, apart from fundamental issues such as randomization and comparison with placebo or a different comparator, the specific statistical design of the trial, e.g. the use of co-primary outcomes or the number of interim analyses, is not very relevant for patient groups to be involved in, although honesty about this to patients who are participating in a trial is very important.

***Quote P3:*** *‘I think most patients have limited knowledge of research designs. I am not sure if patients should be explicitly involved in that. I do think it is a good idea to involve patients in specific questions. Such as, you know, what are the most prominent complaints? Right, so based on that information, I expect that researchers could decide what the optimal outcomes should be in a potential study. But when it comes to design.. well, I don’t think that.. I actually don’t think patients should be directly involved. I do think they should be explicitly informed about each design. It is unacceptable to fool patients. ’*

### Theme 3: Trial participation

#### Preconditions for trial participation

One PTT-member mentioned that she would have liked to know more about clinical trials before she was taken to the hospital and found herself confronted with the choice whether or not to take part in a trial. It should be more common knowledge, also for healthy people, that trials are often performed in hospitals in order to generate evidence about treatments.

***Quote P10:*** *‘yes, if I was to change something, I would certainly ensure that patients generally have a better understanding of clinical trials. And I mean healthy patients, because nobody knows on what day, or what time of any day that they could be struck by with a very serious acute illness, like I was..’*

Another PTT-member mentioned that it is important that in prospective trials participants are informed clearly about the consequences if they choose to participate a trial. Most informed consent letters or trial brochures do not mention the practical aspects which are after all very relevant for patients.

***Quote P4:*** *‘As a PI [Principal Investigator] you can create a slick brochure and all, or even mention the inclusion and exclusion criteria on internet. But if for example it is not explicitly stated that during the entire trial you are not allowed to go on holiday, then this is useless. And while an investigator.. will probably not think of such limitations, but from a patient’s perspective such limitations are very important.’*

#### Participating in a trial as the only option for treatment

For many patients, specifically patients with a rare disease that is life threatening and/or for which no treatment is available yet, a trial is a source of hope, according to the PTT-members. A trial gives patients the opportunity to receive a treatment that would otherwise not be available to them. When a treatment could mean the difference between life or death, they feel that they have no choice but to participate in trials, and they are willing to accept almost any terms.

***Quote P9:*** *‘it was just an academic experiment.. But we knew exactly what we were doing and we felt that, you know, it was a good choice, because there was no other choice. He was going to die.’*

***Quote P3:*** *‘As a patient, in a way you are dependent and vulnerable. That is of course.. that’s the point. Because everyone also knows that in fact you have little choice. Right, so it is not.. it all sounds very good of course, you have to give consent and all, but actually you have no choice. So you are inclined to consent to practically anything.’*

Most of the PTT-members mentioned that before deciding on participating in a trial, they make a risk-benefit assessment. Does the risk of participating outweigh the possible advantages? These advantages may be individual advantages, but also advantages for the patient group.

***Quote P8:*** *‘Actually, participating in a trial is inherently a benefit risk, you see, it’s also simply a trade-off and you think like.. and it doesn’t even have to be to your own benefit. Because you think: well, this is really worthwhile, this makes sense, this will yield something for the group and I’m not at too much risk, so I think that mostly that will be the most important consideration.’*

A PTT-member mentioned a situation in which a trial was being set up, and people wanted to be in the trial, or they want their child to be in the trial, at all costs. The member argued that this may lead to patients or parents who want to jump the queue or have people included even when this would hamper the trial.

***Quote P9:*** *‘Well.. trials get full very quickly. So.. if you are taking people, because they have been ringing you endlessly and you promised them then you are excluding people who didn’t have that information.. I’m really keen to see.. I would be very pleased to see legislation that did not allow people to hound companies to get into the trial. I actually challenged a patient joining a trial in [country] very recently. The trial is a [national] trial. And it wasn’t to protect patients from [country] as a principle. It was a [national] trial.. it’s a very huge amount of intervention. And the one patient from another country, I’m not going to say which because it was very easy to identify this.. was, you know, somebody who was very formidable in raising money.. had persuaded the company that he.. his child should be put into the [national] trial. But the patients [of the national trial] had already participated in the earlier study, which.. met the criteria. And they were wanting to ditch one patient, to put in another. And I.. intervened. Because we had the best patients, we had a child of 18 months who would probably be a best responder. And the child the father wanted to parachute in, was least likely to respond.’*

#### Communication between patients

The PTT-members often mentioned that in their patient communities, patients or their parents communicate with each other, through social media such as Facebook or when they meet at conferences or in a hospital. Both positive and negative aspects of this communication were identified. A positive aspect was that it may help build a patient community when the patients meet or talk to each other online regularly. On the other hand, patients may, during a trial, also discuss the treatment that they have been given. According to one of the PTT-members, this may cause negative feelings in patients who have other, possibly less positive results from their treatment. It may also hamper the double blind standard of placebo controlled trials; PTT-members warned us that patients or parents of patients may find out who has been assigned to the experimental intervention or to the placebo arm. This concept of ‘breaking blinding’ or ‘unintentional unblinding’ may also be known in other areas outside rare diseases where patients are involved and their community is close.

***Quote P8:*** *‘Yet at the same time there are parents who in a manner try to unblind the study, so to speak. One might say: yes, but my child has spots where he was injected and yours doesn’t, so probably yours isn’t and mine is.. well, those kinds of things. Others will check the urine sticks to see if there is protein in the urine. They might then say: yes, my child has a bit of protein in the urine and yours doesn’t. Or the other way around. So.. and we also know of a study where, like, the children who were treated and the children who were in a placebo group were in the same doctor’s office while they were being dosed, subcutaneously, so that’s not very convenient either. You know, people will compare. So actually you do want to share general information, but on the other hand you don’t want them to pay too much attention to each other, so to say, and thereby unblind or even influence the study in such manner.’*

#### Invasive procedures

During a trial, some of the measurements that are performed on patients are more intrusive than others. For example, a biopsy is more intrusive than a questionnaire. According to the PTT-members, it is important to consider the burden for the patients who take part in the trial. When intrusive measurements such as biopsies are included in a study, these measurements have to be very relevant to the study.

***Quote P8:*** *‘And in most trials there have initially also.. been biopsies taken of muscle tissue. And actually muscle biopsies are.. well.. actually of course not very pleasant for children, but it turns out that in the end, they’re performed simply for proof of principle.. and it is quite reprehensible, that their usage is so limited [..]’*

### Theme 4: Phase after the trial

#### Collaboration with regulators and HTA authorities

One of the topics that was regarded as very important by some of the PTT-members was their collaboration with regulators. Sometimes there is a discrepancy between authorities such as the European Medicines Agency and Health Technology Assessment (HTA) authorities or health care payers in individual countries, who look for different types of evidence, which was raised as a problem by some of our PTT-members. However, we have considered this topic beyond the scope of this paper, as it is more concerned with access to medication than with clinical trial design.

#### Information after trial has ended

The final topic that was mentioned is the quality of communication between researchers, clinicians, and pharmaceutical companies on the one hand and patients on the other hand, particularly after a study has ended. Results of a study should be communicated to patients clearer and more often.

***Quote P10:*** *‘maybe things have changed now, in terms of the amount of information that is provided to patients. You know, further down the line, so once the trial closes, I don’t think that there’s a huge amount of information provided to patients.’*

## Discussion

In this study we tried to identify what are the most relevant topics for patients regarding trial design in rare diseases. We have found some general, and some more specific topics within four themes.

In general, it can be concluded from this study that for many rare disease patients, taking part in a trial is not an univocal decision. In the literature, for non-rare diseases most often altruism is mentioned as the main reason for participation in a clinical trial [22]. However, for many rare diseases a treatment is still beyond reach, and beside altruism, a chance to receive an experimental treatment may be a driving factor in rare disease patients. When there are no treatments available, a trial is often the only hope. This means that sometimes, patients are willing to risk anything to participate, even when the chance of getting any personal benefit from the trial is relatively small.

Because for many rare disease patients a trial is a source of hope, many want to decrease the chance of being allocated to the placebo arm of a trial as much as possible. This statement, although understandable from a patient, or patient parent’s perspective, is at odds with the principles of randomized clinical trial design. One of the suggestions made by our PTT-members, to diminish the need for placebo comparison, without decreasing the comparison with a placebo, was to start with collecting patient data in a registry, when no drug trial is in the pipeline yet. Such a registry could provide information on the patients not under treatment, that might be helpful in reducing the placebo group at a later stage. However, such a design does not include randomization. Additionally, the cross-over study design was mentioned as an option. Although such a design is a good way to divide the possible negatives of both trial arms equally over all patients involved, a cross-over design is only possible in very specific circumstances [23].

There are several other topics that are important to patients. One of the topics that patients are specifically interested in, is the choice of outcome measures. No one can assess the clinical relevance of an outcome measure better than those who live with the consequences of a disease on a day to day basis. Therefore, patient involvement in the choice of outcome measures should be the default. This is also in accordance with the work done by the IRDiRC taskforce on Patient-Centered Outcome Measures [24]. This task force identifies the needs to develop outcomes that are relevant to patients, to improve the quality and feasibility of rare disease trials. Also, we recommend that patients are involved in the decision on study length, the target population and the type of information that is provided to potential participants. The input of patients can be useful in for example editing informed consent forms or information leaflets to make them more comprehensible and concrete for patients.

In the collaboration between patients and researchers (pharmaceutical or academic), one of the most important factors is communication. Patients want to be kept informed, and want to know what the pros and cons are of participating in a trial. When patients and researchers work together from the first stages of trial design on, they level more equally with researchers. More experienced patient representatives have an insight in what aspects of a trial are relevant, based on previous trials or experience. They should also be involved in the design phase when decisions are made about (possibly invasive) measurement procedures because they have an idea of which procedures may not be accepted by potential participants. Such a collaboration may also help researchers to recruit and retain enough participants in a later stage. Collaboration between patients and researchers has already shown its value in many research areas [25–28].

The main strength of this study is the qualitative nature of the study design. All topics that are covered in this paper were raised by the participants, who all have some personal or professional experience with trials in the setting of rare diseases. Since the group of patient representatives that we asked to participate in this study is the Patient Think Tank of a European research project (the ASTERIX project), the participants and the interviewers had a good understanding and could rely on an already existing relationship. Each interview was conducted by two interviewers and recorded, which was helpful in making the interviews smooth, efficient and comprehensive. All participants reviewed and approved the transcript of their interview, as well as a draft version of this manuscript.

There are also some limitations to this study that should be considered. Firstly, the group of participants is a convenience sample, and cannot guarantee knowledge saturation. On the other hand, the participants were selected to be part of the Patient Think Tank based on their experience but also taking into account the variety of rare diseases that they represent. Therefore, the group was a balanced mix between acute and chronic diseases, and rare and ultra-rare diseases, and both patients and parents of rare disease patients were present. Also, they represented ten different patient organizations in several countries in Europe. Nonetheless, a group of ten is probably too small to reach data saturation, and cannot guarantee that all opinions are documented. Secondly, for some participants it was not possible to take part in the interview in their native language. However, the level of English of the participants was very high, as can be expected of a group that takes part in a Patient Think Tank in a European project. For practical reasons the interviews were conducted during video-conferencing, which might have been a drawback if the interviewers and PTT-members had not known each other beforehand.

## Conclusion

Many topics that were raised by the participants may improve collaboration between patient groups or patient representatives and researchers in general. Most of our research findings do not apply to rare diseases only. We recommend that researchers and patients, patient organizations or patient representatives collaborate from an early stage on, as such a collaboration will work both ways: it may help to improve empowerment in patients, while simultaneously creating a more feasible design and possibly better recruitment rates.

## Summary

The main topics and recommendations that were derived from this study are:

- Involve patient groups from an early stage on
- Involve patients at least in the following topics:
  - ○ Choice of outcomes and measurements
  - ○ Study duration
  - ○ In- and exclusion criteria
  - ○ The information provided to potential participants
- Consider minimal allocation to placebo, e.g. by using cross-over designs, historical data from a registry or by the use of a common placebo arm in several trials Start a registry as soon as possible, preferably in close collaboration with a patient group
- Educate the wider audience on clinical trials
- Be cautious when assuming that double blind studies remain blind in patient groups
- Keep patients informed after the trial has ended

## Additional file

Additional file 1: Interview guide. (DOC 24 kb)

## Abbreviations

ASTERIX: Advances in Small Trials dEsign for Regulatory Innovation and eXcellence
HTA: Health Technology Assessment
PTT: Patient Think Tank
RCT: Randomized Controlled Trial
