# Supplementary material for: The patient’s view on rare disease trial design – a qualitative study
Source: Orphanet J Rare Dis. 2019 Feb 7;14:31. doi: 10.1186/s13023-019-1002-z (PMC6367834; doi:10.1186/s13023-019-1002-z)
Supplement: Supplementary file 1 — Interview guide. (DOC 24 kb) [file 13023_2019_1002_MOESM1_ESM.doc]

**Additional file 1: interview guide**

Before every interview, one of the interviewers read the following text to the participant:

**“Thank you for your willingness to participate in this interview. We would like to ask you a few questions about your personal experiences in medical research. We want to limit this to the rare disease for which you were asked to be a member of the Asterix Patient Think Tank, which is …. Topics that we would like to discuss are your experiences in medical research on rare diseases, what type of research this was, how you were involved in this research, and what aspects of research and research design you consider important. Do you think it is OK if this conversation is recorded?”**

**“In this interview, we want to collect your experiences, and those of other patients or their caretakers, and subsequently describe all experiences in a structured way. Eventually, we want to write a paper in which the experiences of all participants are documented in an anonymized way. We will do our best to get this paper published in an open access journal, so that not only the members of the ASTERIX consortium, but also other researchers will be able to read your experiences. Do you agree with this?”**

During the interview, we asked the patient representative about his or her experiences regarding trials and trial design. To get a complete picture, the following questions were used as a basis:

1. *What condition do you or your son/daughter have?*
2. *What drugs are currently available? What is your experience with those drugs?*
3. *Can you tell us something about your experiences with medical research as a patient or parent of a patient? What were the positive and negative aspects?*
   1. *What was the treatment like of this trial? Did you know if you had a chance of getting a placebo? How long did the trial continue? How did the researchers measure if the treatment had any effect?*
   2. *What were your circumstances like when you entered this trial? Why did you choose to participate in this specific trial?*
   3. *What problems have you encountered during this research? What aspects did go well?*
   4. *Did you have any contact with other patients or parents during that trial? What was that contact like?*
4. *What aspects do you perceive as most important in a trial?*
5. *Have you ever been approached for a trial that you decided not to participate in? Why did you decide not to participate?*
6. *What kind of research would you like best? What would your ideal type of research look like?*

These questions were not asked literally to the participants, but were used as a guideline to the interviewers. The participants were also further interrogated on topics that they came up with during the interview.
